# The active cohort: a population-based smartphone intervention for health outcomes

**DOI:** 10.1093/pubmed/fdaf090

**Published:** 2025-07-29

**Authors:** Adi Berliner Senderey, Tom Mushkat, Ofer Hadass, Daphna Carmeli, Eyal Jacobson, Aiden Doherty, Derrick A Bennett, Ran D Balicer, Samah Hayek

**Affiliations:** Clalit Research Institute, Innovation Division, Clalit Health Services, Ramat Gan, 6578898, Israel; The Ivan and Francesca Berkowitz Family Living Laboratory Collaboration at Harvard Medical School and Clalit Research Institute, Boston, MA, 02115, USA; Clalit Research Institute, Innovation Division, Clalit Health Services, Ramat Gan, 6578898, Israel; Psychology Department, Social Science Faculty, The Hebrew University of Jerusalem, 91904, Jerusalem, Israel; Clalit Supplementary Health Services, Clalit Health Services, 6116201, Bnei Brak, Israel; Department of General History, Faculty of History, University of Haifa, 3498838, Haifa, Israel; Clalit Supplementary Health Services, Clalit Health Services, 6116201, Bnei Brak, Israel; Clalit Supplementary Health Services, Clalit Health Services, 6116201, Bnei Brak, Israel; Nuffield Department of Population Health, University of Oxford, 30054, Oxford, UK; Nuffield Department of Population Health, University of Oxford, 30054, Oxford, UK; Clalit Research Institute, Innovation Division, Clalit Health Services, Ramat Gan, 6578898, Israel; School of Public Health, Faculty of Health Sciences, Ben Gurion University of the Negev, 8410501, Be’er Sheva, Israel; Clalit Research Institute, Innovation Division, Clalit Health Services, Ramat Gan, 6578898, Israel; Department of Epidemiology and Preventive Medicine, School of Public Health, Gray Faculty of Medicine, Tel Aviv University, 69978, Tel Aviv, Israel

**Keywords:** behavior, epidemiology, public health

## Abstract

**Background:**

The Clalit Active Cohort Study (CACS) assess the impact of lifestyle factors, particularly physical activity, on short- and long-term health outcomes using real-world data. Launched in January 2021, CACS focuses on Clalit Health Services members with supplemental health insurance who use the Clalit Active smartphone app.

**Methods:**

The study integrates data from the Clalit Active app with electronic health records from CHS, covering primary and secondary care, hospitalizations, medications, laboratory results, and imaging. The cohort currently includes 622 584 participants and continues to grow.

**Results:**

The app monitors various health-related behaviors, including physical activity and sleep. Preliminary findings show significant variations in daily step counts based on sociodemographic and clinical factors. Substantial differences were found between app users and non-users app users and non-users. On average, males recorded higher daily step counts compared to females, and individuals under the age of 40 demonstrated greater activity levels than older participants. Participants with pre-existing comorbidities demonstrated lower activity levels.

**Conclusions:**

CACS is a powerful resource for researchers and policymakers, providing insights into the relationship between lifestyle factors and health outcomes within a diverse population. Findings can inform public health policies and guide the lifestyle interventions, highlighting the potential of integrating smartphone data with electronic health records to improve health outcomes.

## Introduction

The health benefits of physical activity have been extensively studies, showing that being engaged in physical activity is associated with a substantially decreased risk of premature mortality, chronic conditions (e.g. cardiovascular disease, diabetes, or depression), and specific types of cancer.^1–5^ However, the adherence rates to regular physical activity remain low.

In the United States, ~8% of adults fail to meet the regular guidelines, which suggest 150 min of moderate-intensity physical activity per week.^6^ Similarly, in Europe, where the World Health Organization (WHO) recommends at least 30 min of moderate-intensity physical activity 5 days a week or at least 20 min of vigorous-intensity physical activity 3 days a week, about one in three adults remains insufficiently physically active.^7^

With the increasing availability of smartphones and digital health technologies, and the growing burden of chronic conditions at younger ages, there is a timely and scalable opportunity to integrate digital tools into physical activity promotion strategies.^8^ Previous studies focusing on the effectiveness of physical activity have incorporated behaviorally designed interventions to promote increased physical activity by using monetary rewards, psychological and social incentives.^9–12^ Most of these studies are limited by design, since they rely on the combination of few randomized controlled trials, self-reported information, or qualitative studies.^13^

To date, few studies have described smartphone-based interventions to enhance physical activity at a population level or within a real-world healthcare setting. The Israeli national physical activity guidelines are consistent with the WHO recommendations, advocating for 150–300 min of moderate-intensity or 75–150 min of vigorous-intensity per week.^6^^,^^14–16^ However, a national survey revealed that only 29.1% of Israeli adults (age ≥ 21 years) met these guidelines between 2018 and 2020.^17^ The Clalit Health Services (CHS) has identified a need for promoting healthy lifestyle through new technologies and launched the Clalit Active app as a population-based intervention for Clalit members.

This study aims to **describe the Clalit Active Cohort Study (CACS)** and **its utilization** among CHS members, focusing on how the Clalit Active app is used to monitor and promote physical activity. This study provides a comprehensive overview of the CACS, including its integration with electronic health records (EHRs) and the types of data collected. By detailing the cohort and its data infrastructure, we aim to illustrate the potential of smartphone-based health interventions in a large healthcare system. The CACS serves as a data repository on lifestyle factors and health outcomes, allowing researchers to explore patterns of physical activity and health within a large, diverse population. This descriptive study lays the groundwork for future research on the impact of such interventions and highlights the utility of integrating digital tools within healthcare systems.

## Methods

### Study design and population

The CACS is an ongoing prospective cohort study that collects data on a daily basis.

CACS, strategically positioned within CHS, the largest of four integrated healthcare organizations in Israel. With a vast reach covering more than 4.7 million members, constituting 53% of the Israeli general population, CHS provides a comprehensive platform for health services. This study includes a subset of CHS members, those with supplemental health insurance (SHI) and access to the Clalit Active app, consisting of 80% of CHS’s members. Clalit’s comprehensive healthcare data warehouse was digitized at the start of the millennium and combines hospital and community medical records. Clalit EHRs contain administrative and clinical data, sociodemographic information, diagnoses from community and hospital settings, chronic diseases, and biomarkers.^18^ Chronic conditions within EHR are coded according to the International Classification of Diseases, 9th revision (ICD-9: International Classification of Diseases, Ninth Revision).

Eligible participants were CHS members aged 16 years or older with at least 1 year of continuous membership in CHS and who held supplemental health services coverage. Participants needed to have registered to use the app between January 2021 and May 2023. We excluded individuals who did not complete the onboarding questionnaire, CHS healthcare workers (i.e. due to their selective behavior), members without 1 year of CHS membership, and those marked as bed-ridden.

App users were defined as those who downloaded the app, completed the baseline questionnaire, and logged into the app at least three times within the first 30 days, indicating meaningful engagement during the initial month of use. All other eligible members, who did not download the app, were categorized as non-users. The index date was defined as the date on which participant downloaded the app for the app users, and the data extraction date (10 May 2023) for the non-app users. Participants who subsequently stopped using or engaging with the app during the follow-up period were classified as having experienced attrition. (Appendix Fig. S1—flow chart of study participants).

### Data collection and management

At the time of using the App, each participant’s data is linked with Clalit EHR data retrospectively. The uploaded data are transferred and integrated with data repositories coming from Clalit EHR on a daily basis. This interoperability between app logs and health records facilitates the longitudinal integration of medical information, continually updated physical activity and lifestyle-related data for all Clalit Active users. Appendix 1 provides a list of the Clalit Active App core variables (Appendix Table S1).

The Active App communicates and interacts with its users, encouraging them to increase their physical activity and reach weekly personal health goals. These goals constantly evolve based on user activity and informed health parameters for each individual. The App has an ongoing incentive system. The incentives were devised based on behavioral science methods, including personalization based on individual performance and preferences.^9^^,^^11^^,^^13^ These data-driven incentives are designed around financial, psychosocial, and cognitive aspects to encourage participation and promote higher adherence to individual goals, thereby reducing loss to follow-up. By offering personalized incentives tailored to each user’s physical activity levels—encompassing psychological, social, and economic dimensions—the system reinforces sustained positive behavior by aligning with users’ motivations and preferences. A detailed description of the incentives is described in Appendix Table S2.

All members of CACS members are required to complete a comprehensive self-report questionnaire upon downloading the app. This baseline questionnaire captures a wide range of data, including attitudes, toward health, health-related habits (e.g. smoking, sleep), weight, and physical activity levels. This initial self-reporting is supplemented by continuous, real-time data collection through the Clalit Active app, which objectively records daily metrics such as the number of steps taken, duration and intensity of physical activity, sleep duration, and self-reported dietary habits and hydration levels. The app also captures subjective health awareness, allowing for a nuanced understanding of health behaviors and perceptions. Appendix Fig. S3 describes a screenshot from different data collected from the App.

A key innovation of the CACS lies in the real-time dynamic integration of these data streams with Clalit’s comprehensive EHRs, the largest health maintenance organization in Israel. This linkage is performed daily using unique personal identifiers, ensuring real-time integration of step count data with rich clinical information, including diagnosis (e.g. cardiovascular disease, diabetes, and hypertension based on ICD-9 codes), laboratory results, and medication use, are seamlessly integrated. This continuous data integration enables robust, population-level analyses of health behaviors and outcomes, supporting exploration of potential causal relationships in a way that were previously unattainable.

All data are securely stored within Clalit’s infrastructure, adhering strictly to national privacy regulations and ensuring the confidentiality and integrity of participants’ information.

### Study period

Given that the CACS is an ongoing cohort study, the data and analysis presented in this manuscript are based on information collected between January 2021 and May 2023. This study focuses specifically on the demographic and clinical characteristics of participants at index date.

For consistency and improved data visualization, the analyses for the figures only were limited to a maximum of 700 days. Beyond this period, step count data became increasingly sparse, resulting in unstable estimates and greater variability. As a result, a 700-day threshold was set to cap the follow-up period for analyses involving step-based metrics (Appendix Table S2).

### Statistical analysis

Descriptive statistics were used to characterize the study population. Chi-square test and *t*-tests were conducted to compare between users versus non-users. Multiple logistic regression models were used to assess the association between chronic conditions and app usage. Results are presented as odds ratios (ORs), with 95% confidence interval (95% CI) to quantify the strength of the associations. All models were adjusted for age at baseline, sex, socioeconomic status, and ethnicity. Analysis were performed using R software.

### Ethical considerations

All the participants signed separate informed consent for data use at the time of downloading the app. Ethical approval for this study was provided by the IRB of the CHS organization Committee (0068-21-COM1).

## Results

By January 2021, 3 347 258 CHS members were considered eligible for CACS, 622 584 of whom downloaded the app and completed the baseline questionnaire by May 2023 and 1 767 708 were non-users (Appendix Fig. S1). During the study period on average, each month 26 138 participants is enrolled in the cohort. Participants have consistently used the App with a ~25% attrition rate. Each participant has access to full information about their interaction with the App as well as their medical record data.

The sociodemographic and clinical characteristics of the cohort participants (i.e. Clalit Active and non-Clalit active) is presented in Table 1. Compared to non-active app users, the active app users were more likely to be female (59% vs. 51%), Jewish (88% vs. 74%), be younger (average age 42, (SD: 16) vs. 47 (SD: 20), have higher socioeconomic status (29% vs. 23%), and have lower rates of chronic conditions (i.e. 5% vs. 10% with diabetes, 12% vs. 21% with hypertension, etc.) (Table 1). In adjusted analysis, patients with different chronic conditions were less likely to be app users. For example, CVD patients had 27% lower likelihood to be app users (OR: 0.73; 95% CI: 0.72–0.74) (Table 2).

**Table 1:** Sociodemographic and clinical characteristics of the study population.

Characteristic	App users	Non-users	*P*
*N*	622 584	1 767 708	
**Sociodemographic characteristics**			
Age, years, mean (SD)	42 (16)	47 (20)	<.001
Female	368 185 (59%)	894 400 (51%)	<.001
High socioeconomic status^a^	177 614 (29%)	402 615 (23%)	<.001
Jewish	546 818 (88%)	1 306 144 (74%)	<.001
**Lifestyle behavior**			
Smoker	86 144 (14%)	306 445 (17%)	<.001
Obesity (BMI ≥ 30)^b^	132 792 (21%)	420 770 (24%)	<.001
**Physical measurements**			
HbA_1c_ (%), mean (SD)^c^	5.91 (1.08)	6.07 (1.25)	<.001
Glucose (mg/dl), med (IQR)	90 (14)	91 (17)	<.001
HDL (mg/dl), mean (SD)	52 (13)	51 (13)	<.001
LDL (mg/dl), mean (SD)	103 (31)	101 (33)	<.001
Triglycerides (mg/dl), med (IQR)	96, (68)	101 (70)	<.001
**History of chronic disease**			
No. GP visits in 5 years,^d^ med (IQR)	30 (34)	32 (40)	<.001
Cardiovascular disease	34 479 (5.5%)	203 232 (11%)	<.001
Diabetes	31 356 (5.0%)	178 856 (10%)	<.001
Diabetes complications	14 223 (2.3%)	106 370 (6.0%)	<.001
Hypertension	74 555 (12%)	374 040 (21%)	<.001
Chronic renal disease	7817 (1.3%)	51 155 (2.9%)	<.001
Asthma	12 599 (2.0%)	39 028 (2.2%)	<.001
Bronchiectasis	426 (<0.1%)	2627 (0.1%)	<.001
Pulmonary fibrosis	20 (<0.1%)	186 (<0.1%)	<.001
Chronic kidney disease	1792 (0.3%)	13 890 (0.8%)	<.001
Hyperlipidemia	142 515 (23%)	561 223 (32%)	<.001

*Notes*: All categorical variables were described as *N* (%) and continuous variables were described as mean (SD) for normal distributions or median (IQR: Inter Quartile Range) for skewed distributions.

a Socioeconomic status is based on place of residence (at the level of a neighborhood or a small town).

b BMI is calculated as kg/m^2^.

c HbA_1c_, glycated hemoglobin.

d No. GP visits in 5 years: The number of general physicians’ visits in the last 5 years prior to index date.

**Table 2:** Multiple logistic regressions for the association between chronic conditions and app users.

	OR	95% CI
CVD	0.73	0.72–0.74
Diabetes	0.77	0.76–0.78
Diabetes complications	0.61	0.60–0.62
Hypertension	0.83	0.82–0.84
COPD	0.61	0.59–0.62
Asthma	0.89	0.88–0.91
Bronchiectasis	0.62	0.56–0.69
Pulmonary fibrosis	0.54	0.33–0.84
CKD	0.6	0.57–0.63
Hyperlipidemia	0.94	0.63–0.94

*Notes*: Logistic regression models were performed. OR: odds ratio. 95% CI: 95% confidence interval. CVD: cardiovascular disease.

**Figure 1:**
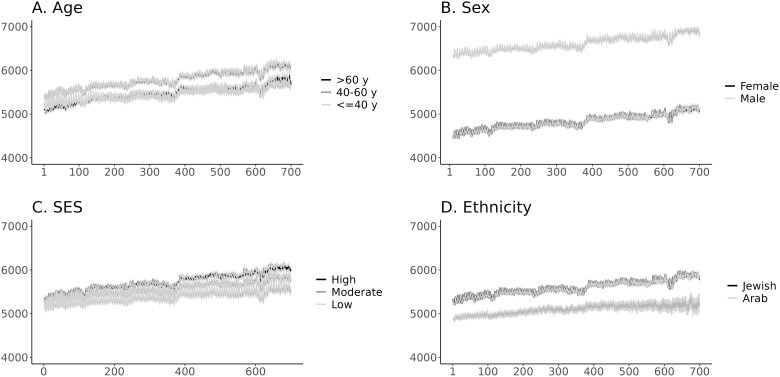
(A: Age) Average daily steps and 95% confidence intervals over research period stratified by patients’ age groups. (B: Sex) Average daily steps and 95% confidence intervals over research period stratified by patients’ sex. (C: SES) Average daily steps and 95% confidence intervals over research period stratified by patient’s SES. (D: Ethnicity) Average daily steps over and 95% confidence intervals over research period stratified by patients’ ethnicity.

The geographical distribution of Clalit Active users is described in Appendix Fig. S3. The heat map shows that the most users are located in the center of Israel (≥250 000 users), which is the most urban district in Israel. The distribution of the number of daily steps across different demographic characteristics is presented in Fig. 1 by age groups, gender, ethnicity, and socioeconomic status. A modest increase in the average number of daily steps over time was observed across all demographic characteristics. After 700 days of follow-up, individuals aged 40–60 had a higher average daily step count compared to those aged 40 years or younger and those aged 60 years or older (Fig. 1A). There was a clear discrepancy in the number of daily steps by gender; males had a higher initial number of daily steps on average compared to females (Fig. 1B). However, both males and females displayed a similar upward trend in daily step count during the follow-up period. Furthermore, there is a difference in the daily number of steps by socioeconomic status (Fig. 1C). Those with a higher socioeconomic status had a higher number of daily steps on average over the study period, followed by moderate and low socioeconomic status. Figure 1D showed differences in the number of daily steps based on ethnic group. The Jewish population group reported a higher initial number of steps on average compared to the Arab population group. Nonetheless, both groups demonstrated similar patterns of increase in daily steps over time during the follow-up period.

**Figure 2:**
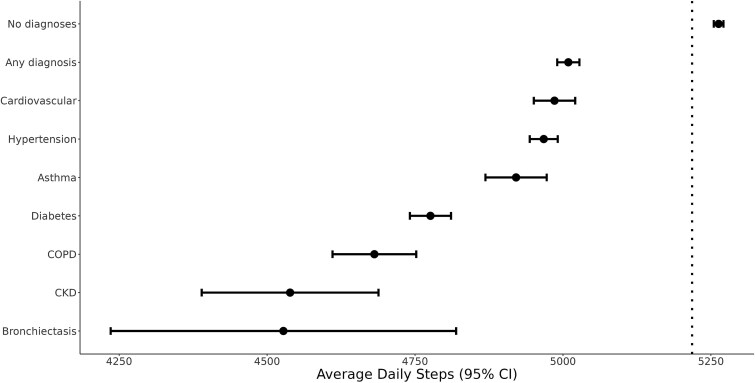
Average daily number of steps for Clalit Active users with and without chronic conditions at baseline. The dotted line represents the average number of daily steps across the entire population.

For users with and without chronic conditions at baseline, the overall average number of daily steps was 5218 (Fig. 2). Users with no prior history of chronic condition at baseline had a higher number of steps per day on average (average of 5263 steps, 95% CI: 5255–5271) compared to users with any chronic condition (average of 5009 steps 95% CI: 4990–5028). The specific diseases with the lowest number of daily steps include individuals with Bronchiectasis (average of 4528 steps 95% CI: 4236–4819), chronic kidney disease (average of 4539 steps 95% CI: 4389–4688), chronic obstructive pulmonary disease (average of 4681 steps 95% CI: 4611–4752), and Diabetes (average of 4776 steps 95% CI: 4741–4811).

**Figure 3:**
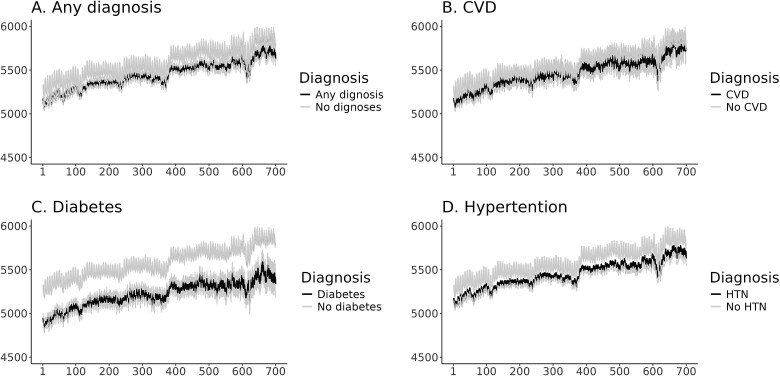
(A: Any diagnosis) Average daily steps and 95% confidence intervals over research period stratified by patients’ who suffered at the study onset from either CVD, diabetes, or hypertension versus patients that did not. (B: CVD) Average daily steps and 95% confidence intervals over research period stratified by patients’ who suffered at the study onset from CVD versus patients that did not. (C: Diabetes) Average daily steps and 95% confidence intervals over research period stratified by patients’ who suffered at the study onset from diabetes versus patients that did not. (D: Hypertension) Average daily steps and 95% confidence intervals over research period stratified by patients’ who suffered at the study onset from hypertension versus patients that did not.

Appendix Fig. S4a and b shows the distribution of average daily number of steps for users with and without chronic conditions at baseline by age and gender. The older age group has lower number of steps, as well females. The distribution of the number of daily steps among the active cohort members by specific chronic conditions at baseline is described in Fig. 3. Figure 3A shows that those who have any chronic conditions at baseline reported a lower number of daily steps across the 700 days of the follow-up; however, the gap was small between the two groups throughout the follow-up. For those with CVD, the difference in the average daily steps was not markedly different compared to those without CVD (Fig. 3B). A similar pattern is seen for hypertension (Fig. 3D). Conversely, the difference in the daily steps between those with and without diabetes was more noticeable (Fig. 3C).

Furthermore, Appendix Figs S5a–d and Figs S6a–d illustrate the pace (defined as the number of steps per minute) of app users based on demographic and clinical characteristics, showing patterns consistent with average step counts. Higher intensity of step counts was seen among younger individuals, males, those with higher socioeconomic status, and those with chronic conditions.

## Discussion

The CACS is a population-based dynamic cohort that is embedded within CHS. The CACS was established as a resource for researchers and policymakers, to allow for the evaluation of both short- and long-term impacts of lifestyle factors on different health outcomes. This cohort provides a unique opportunity to explore the effects of personalized interventions and to monitor trends in behaviors (e.g. physical activity, sleep, etc.) on biomarkers and chronic conditions, all using real-world data.

One of the innovative aspects of the CACS is its integration with the CHS data repository, which enables long-term follow-up of participants, both active and non-active users of the Clalit Active app. The availability of data for both groups allows for comprehensive comparisons. Our initial findings indicate that active users demonstrate healthier lifestyle habits and a lower prevalence of chronic conditions compared to non-active users. Among the active users, an increase in physical activity over time was observed. This increase was particularly notable among those without chronic conditions, who consistently showed a higher average daily step count. This pattern remained consistent across different chronic condition categories. Furthermore, we have seen that patients with chronic conditions are less likely to download the active app and be users compared to patients without chronic conditions, on the otherhood knowing that active patients less likely to develop conditions.^19^ Future research should focus on specific chronic conditions and examine strategies to promote physical activity as a means to reduce complications and adverse events.

To the best of our knowledge, the Clalit Active app is one of the first to be integrated within a large EHR system, allowing for individual-level data linkage between the app and EHR. Despite its relatively recent inception, with only 2.5 years of follow-up data currently available, the cohort is well-positioned. As it matures and the participant pool grows, it will provide valuable insights into the long-term effects of digital health interventions. This will enhance our understanding of how physical activity impacts a wide range of health outcomes over time.

There are some limitations to consider: First, with the app being a relatively new initiative, our current follow-up period is insufficient to assess the long-term effects of the Clalit Active app on health outcomes comprehensively. As the cohort matures, more extensive longitudinal data will become available, allowing for more detailed analyses. Second, in the case where members decide to stop using the App, we are unable to determine their reasons for withdrawal. Third the number of steps that are collected via mobile phones might underestimate the physical activity levels compared to more precise methods like wrist-worn accelerometers.^20^ However, this is consistent across the follow-up and we believe that there is no reason to suspect differential information bias. In the future, we plan to collect and integrate data from wrist-worn accelerometers into the CACS dataset. Fourth, the Clalit Active app does not currently support tracking non-stepping activities such as swimming or cycling. While some wearable devices can capture these types of movements, our dataset is limited to step-based data. We hope that future versions of the app will incorporate expanded activity tracking capabilities. Fifth, at present, the Clalit EHR has limited genomic data, but we plan to link the CACS data with the Clalit biobank data that has recently been established in 2023. Finally, the app is only for those who have SHI so therefore the cohort is not representative of the whole of Clalit members or the general Israeli population, although this is a similar issue within many other cohorts that does not limit their ability to identify risk factor–disease relationships reliably.^21^ Moreover, the integration of longitudinal medical information with real-time physical activity levels and other lifestyle-related data for the entire Clalit Active users will create a valuable resource for future research use.

## Supplementary Material

Supplementary-material_6_9_2025_fdaf090

## Data Availability

The CACS data is not freely or publicly available. However, the founders of the cohort have different collaborations utilizing the cohort data. The founders of the cohort encourage new collaborations related to lifestyle and health-seeking behaviors. Potential collaborators are invited to contact us through our website (https://www.clalit-innovation.org/).

## Notes

**Adi Berliner Senderey,** PhD, Team Lead at Clalit Innovation

**Tom Mushkat,** MSC, Data Scientist at Clalit Innovation

**Ofer Hadass,** PhD, Head of Business Development at Clalit Supplemental Health Insurance

**Daphna Carmeli,** PhD, Head of Clalit Supplemental Health Insurance

**Eyal Jacobson,** MD, MPH, Medical health of Clalit Supplemental Health Insurance

**Aiden Doherty,** PhD, Professor at oxford University

**Derrick A. Bennett,** PhD, Professor at Oxford University

**Ran D. Balicer,** MD, PhD, Full Professor at Ben-Gurion University and Head of Clalit Innovation

**Samah Hayek,** DrPH, Assistant Professor at Tel Aviv University and Senior Epidemiologist at Clalit Innovation
