# Supplementary material for: The active cohort: a population-based smartphone intervention for health outcomes
Source: J Public Health (Oxf). 2025 Jul 29;47(4):765–72. doi: 10.1093/pubmed/fdaf090 (PMC12670004; doi:10.1093/pubmed/fdaf090)
Supplement: Supplementary-material_6_9_2025_fdaf090 [file supplementary-material_6_9_2025_fdaf090.docx]

**The Active Cohort: A Population-Based Smartphone Intervention for Health Outcomes**

**Appendix Tables and Figures**

Appendix Table 1: List of the core variables for the Clalit-Active app collection dataset

Appendix Table 2: Top-10 incentives purchases

Appendix Table 3: Distribution of participant by different time period

Appendix Figure 1: Flow chart of the study population and Cohort Enrollment Process

Appendix Figure 2: Clalit Active app screenshots for different data collected

Appendix Figure 3. Heat map of Clalit-Active users by Israeli districts

Appendix Figure 4: Average daily steps stratified

Appendix Figure 5: Intensity of step counts by demographic characteristics

Appendix Figure 6 Intensity of step counts by clinical characteristics

**Appendix Table 1: List of the core variables for the Clalit-Active app collection dataset**

| **Domain** | **Sub-domain** |
| --- | --- |
| Sociodemographic characteristics | Date of birth |
|  | Gender |
|  | Marital status |
|  | Region |
|  | Weight |
|  | Height |
| Lifestyle behavior | Smoking status |
|  | Alcohol usage |
|  | Physical activity: Number of steps ^†^ |
| Health status | Sleep trend |
|  | Activities of Daily Living |
|  | Self-reported health |
|  | Memory |
|  | Dizziness |
|  | Orientation |
|  | Energy |
| Engagement with the active goals | Number of in-app coins ^†^ |
|  | Number of in-app purchases ^†^ |
|  | Achieving physical activity goals ^†^ |
| Clinical and mental condition^1^ | Anxiety |
|  | Depression |
|  | Cardiovascular disease |
|  | Hypertension |
|  | Chronic kidney disease |
|  | Cancer |
|  | Diabetes |

^1^ Data on clinical and mental conditions are extracted from the electronic medical records
^†^ Data collected passively by the Active-App

**Notes:** Physical activity goals were set based on the individual performance for each week. The criteria for each goal were validated by trained exercise physiologists.

**Appendix Table 2: Top-10 incentives purchases**

|  | Total |
| --- | --- |
| Alternative medicine voucher | 20,038 |
| Lenovo headphones | 17,950 |
| massage tool | 17,935 |
| Running cellphone bag | 16,817 |
| Smart weight | 41,519 |
| Sport bag | 27,511 |
| Sport equipment voucher | 167,207 |
| Xiaomi headphones | 59,403 |
| Xiaomi smart band | 83,038 |
| Yoga mattress | 12,894 |

**Appendix Table 3 : Distribution of participant by different time period**

| N | Number of days |
| --- | --- |
| 78,731 | ≤700 |
| 53,851 | 750 |
| 36,334 | 800 |
| 1,440 | 850 |
| 1,167 | 851+ |

**Appendix Figure 1: Flow chart of the study population and Cohort Enrollment Process**

3,347,258 Participants (Clalit members, ≥16 yr of age) as of the beginning of the Clalit-Active program (January 2021) were included.

694,147 (20.5%) Didn’t have supplementary health insurance

2,653,111 (79.5%) Had supplementary health insurance

CHS Health care workers: 71,481 (2.7%)

Did not have continuous CHS membership: 93,993 (3.5%)

Confined to the home or were nursing home residence: 88,752 (3.3%)

1,767,708 (73.6%) Non Clalit-Active users

**634,609 (26.4%)
 Clalit-Active users**

**Did not meet the onboarding questioner to join the program: 12,025 (1.9%)**

622,584 (98.1%) were eligible to be included in the Clalit-Active Cohort

Figure 1: The study population and cohort enrollment process. Absolute numbers and percentages are shown for each inclusion and exclusion criterion. Members could have had more than one reason for exclusion. CHS denotes Clalit Health Services.

Alt text: Flow chart describing the process of patients’ exclusions from the overall population to the final cohort.

**Notes:** CHS denotes Clalit Health Services.

Absolute numbers and percentages are shown for each inclusion and exclusion criterion. Members could have had more than one reason for exclusion. Further we have 707 those who are early adapters and used the app before 1.1.2021were included

**Appendix Figure 2: Clalit Active app screenshots for different data collected**


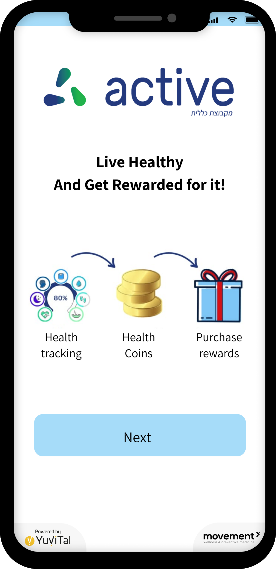

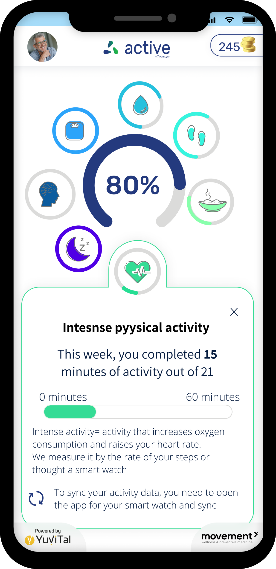

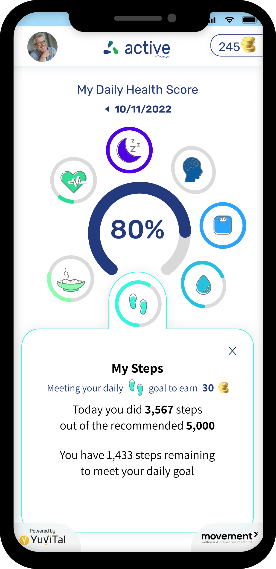

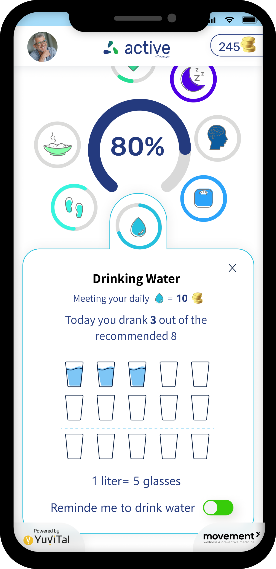

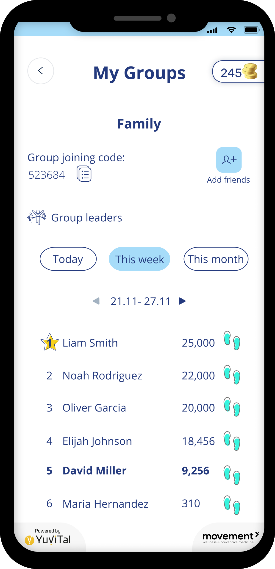

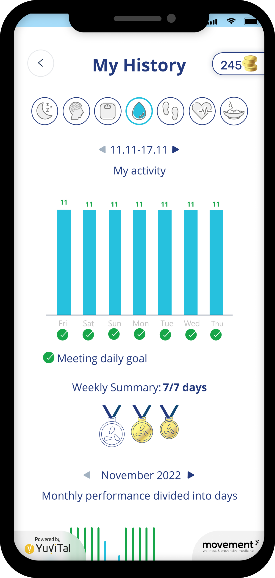

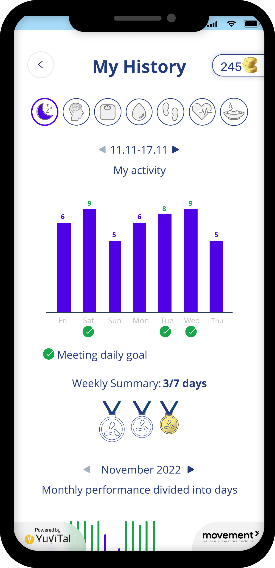

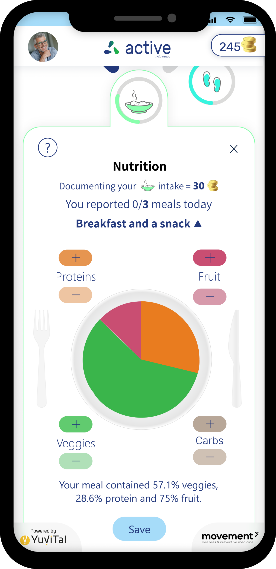


**Appendix Figure 3. Heat map of Clalit-Active users by Israeli districts**

Figure 3: The colours indicate number of Clalit-Active users in each geographical area of Israel


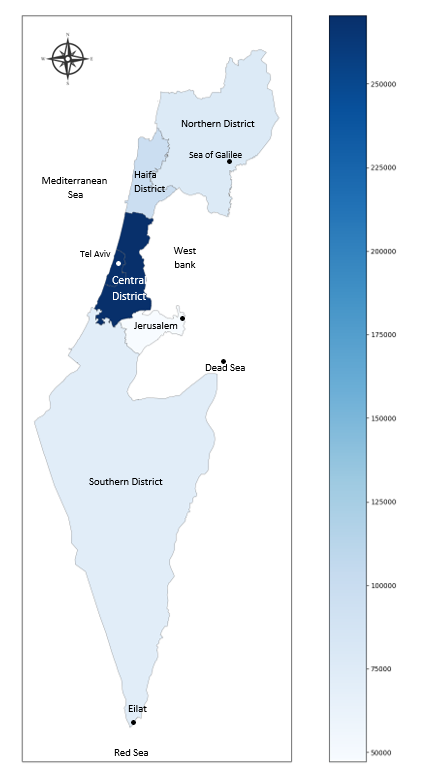


225,000

200,000

175,000

150,000

125,000

100,000

75,000

50,000

250,000

.

**Appendix 4: Average daily steps stratified**

Appendix Figure 4a: Average daily steps by comorbidity


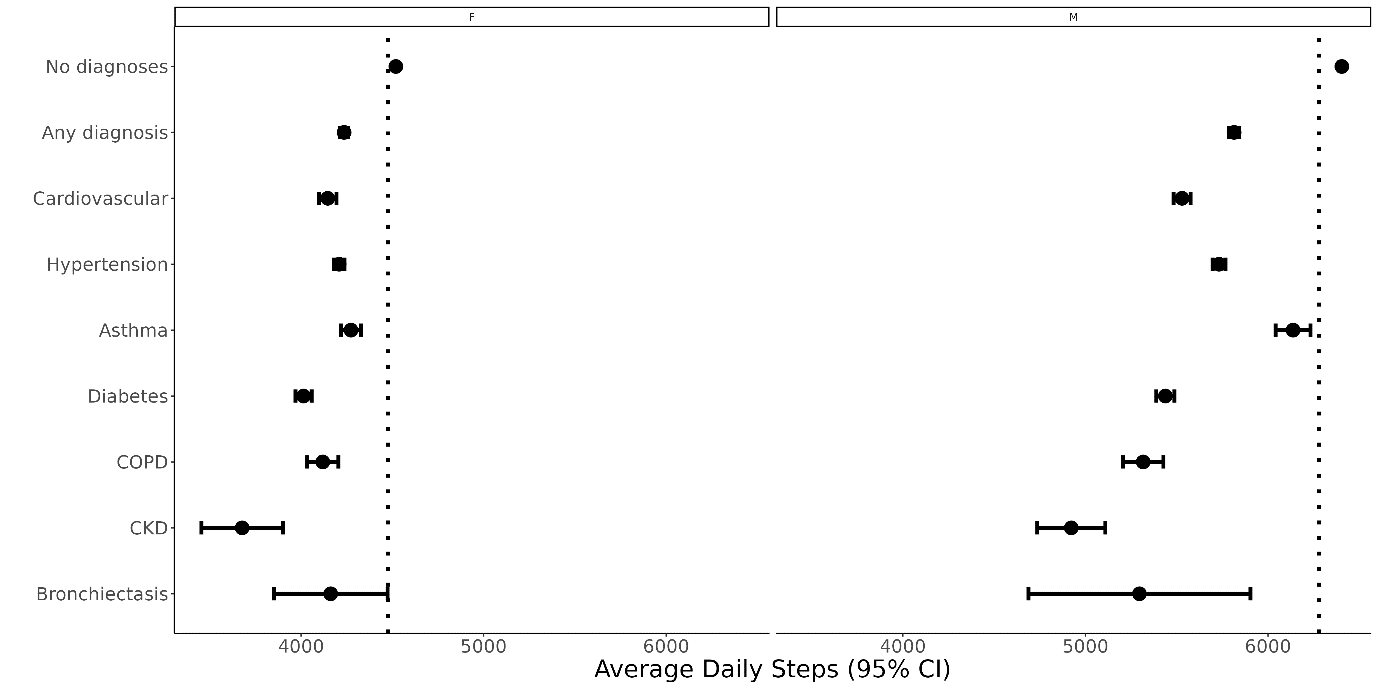


Figure 4.A. Average daily number of steps for Clalit-Active users with and without chronic conditions at baseline stratified by gender. The dotted line represents the average number of daily steps across the entire population. Females are presented on the left figure, and males on the right figure.

Alt text. Figure describing the Average daily number of steps for Clalit-Active users stratified by gender for each subgroup of patients having a specific chronic condition at the study onset. Females are presented on the left figure, and males on the right figure.


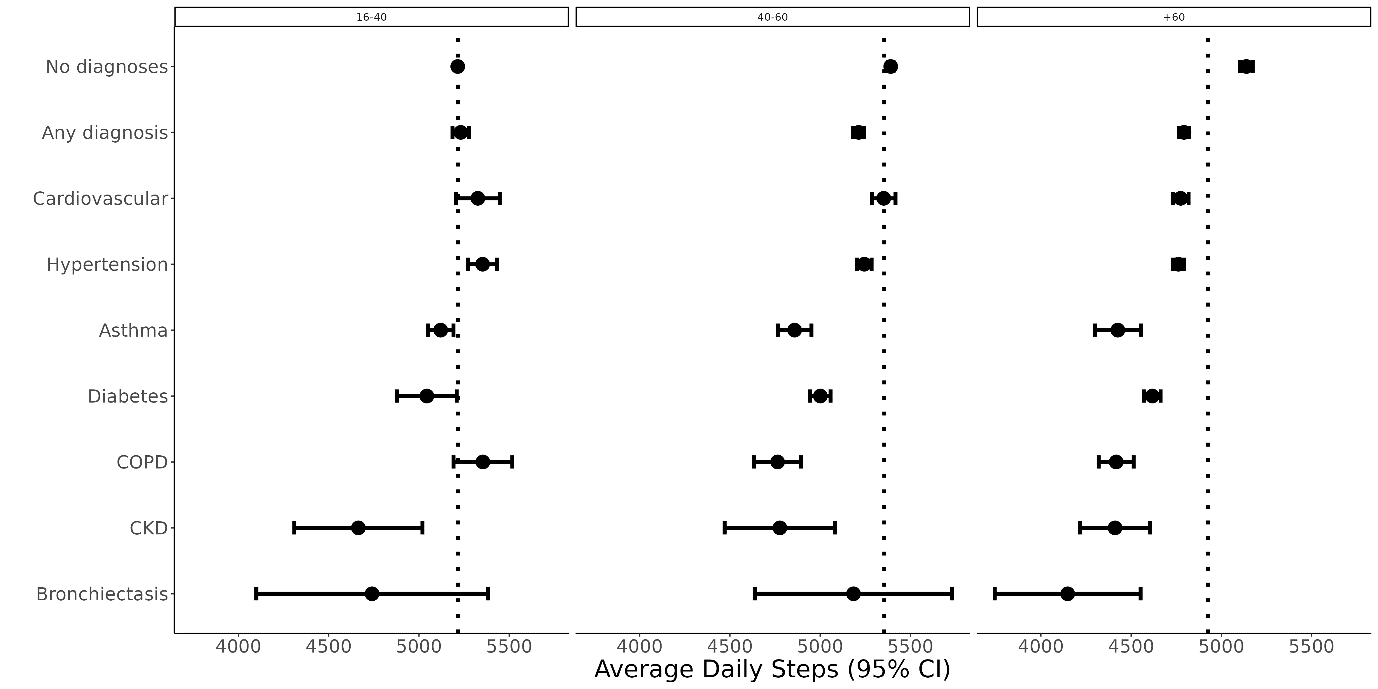


Figure 4.B. Average daily number of steps for Clalit-Active users with and without chronic conditions at baseline stratified by age groups. The dotted line represents the average number of daily steps across the entire population. Members aged up to 40 are presented on the left figure, members aged between 40 to 60 are presented on the middle figure, members aged +60 are presented in the right figure.

Alt text. Figure describing the Average daily number of steps for Clalit-Active users stratified by age groups for each subgroup of patients having a specific chronic condition at the study onset. Members aged up to 40 are presented on the left figure, members aged between 40 to 60 are presented on the middle figure, members aged +60 are presented in the right figure.

**Appendix Figure 5: Intensity of step counts by demographic characteristics**

**
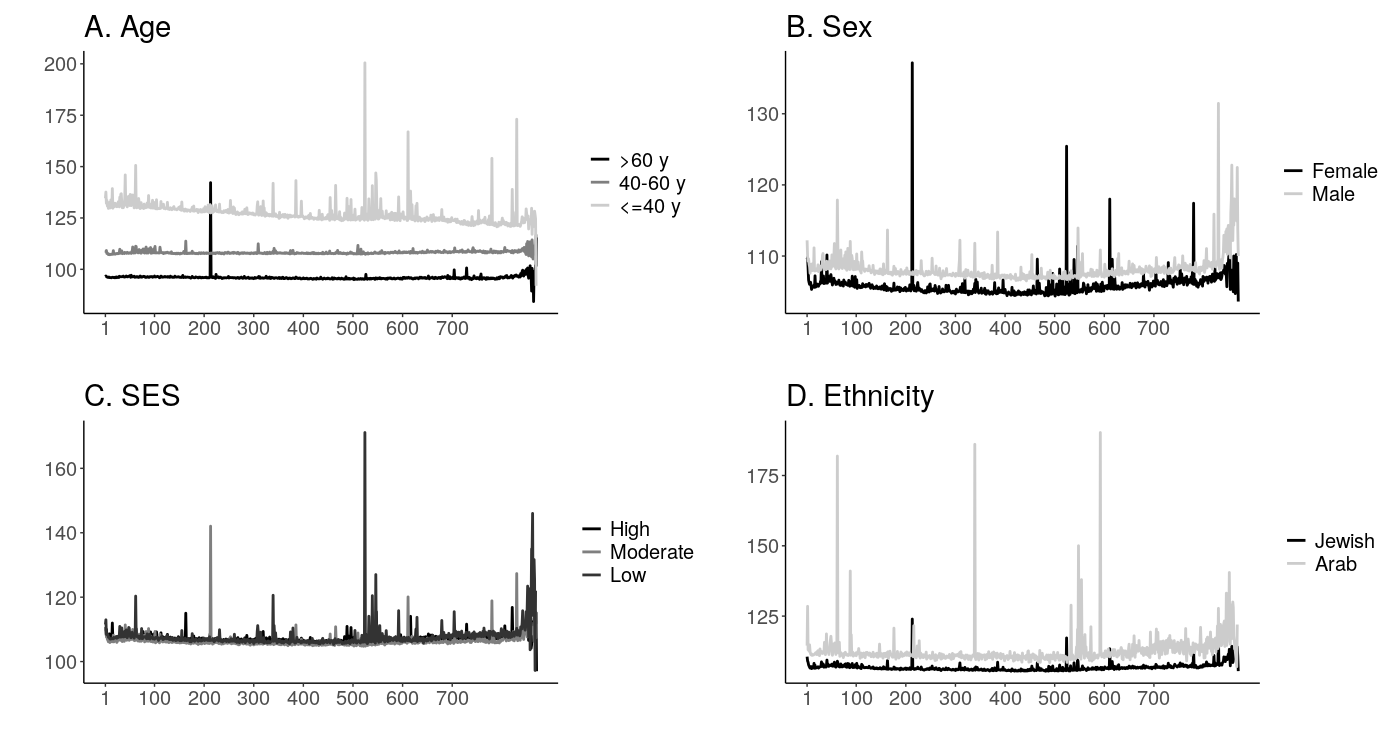
**

Figure 5.A. Average intensity step count for Clalit-Active users by demographic characteristics at baseline over research period. Figure 1. (A. Age) Average intensity step count over research period stratified by patients’ age groups. (B. Sex) Average intensity step count over research period stratified by patients’ sex. (C. SES) Average intensity step count over research period stratified by patient’s SES. (D. Ethnicity) Average intensity step count over research period stratified by patients’ ethnicity.

Alt text. Figure describing the average intensity step count for Clalit-Active users over research period. Each sub figure stratifies the sample by single criterion: age groups, gender, SES, and ethnicity.

**Appendix Figure 6 Intensity of step counts by clinical characteristics**

**
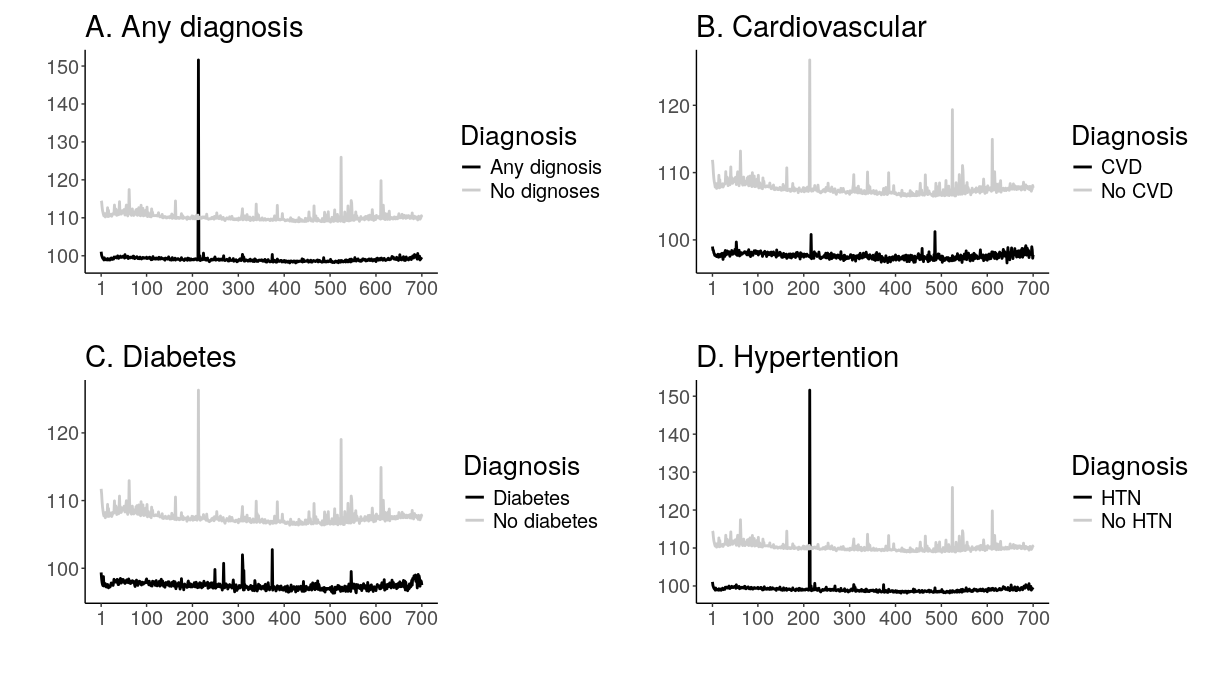
**

Figure 6.A. Average intensity step count for Clalit-Active users by clinical characteristics at baseline over research period. Figure 1. (A. Any diagnosis) Average intensity step count over research period stratified by patients’ diagnosis status. (B. Cardiovascular diseases) Average intensity step count over research period stratified by CVD status. (C. Diabetes) Average intensity step count over research period stratified by patient’s diabetes status. (D. hypertension) Average intensity step count over research period stratified by hypertension status.

Alt text. Figure describing the average intensity step count for Clalit-Active users over research period. Each sub figure stratifies the sample by single chronic condition: having any diagnosis, CVD, diabetes, and hypertension.
